# Mutation identification and prediction for severe cardiomyopathy in Alström syndrome, and review of the literature for cardiomyopathy

**DOI:** 10.1186/s13023-022-02483-7

**Published:** 2022-09-15

**Authors:** Savas Dedeoglu, Elif Dede, Funda Oztunc, Asuman Gedikbasi, Gozde Yesil, Reyhan Dedeoglu

**Affiliations:** 1 Department of Pediatrics, Uskudar University, Istanbul, Turkey; 2 Department of Pediatric Cardiology, Cerrahpasa Medical School, Istanbul University-Cerrahpaşa, Istanbul, Turkey; 3 Division of Medical Genetics, Department of Pediatric Basic Sciences, Institute of Child Health, Istanbul University, Istanbul, Turkey; 4 Department of Medical Genetics, Faculty of Medicine, Istanbul University, Istanbul, Turkey

**Keywords:** Alström syndrome, Severe cardiomyopathy, Genetic mutation

## Abstract

**Objective:**

Alström syndrome (ALMS) is a rare autosomal recessive genetic disorder that is caused by homozygous or compound heterozygous mutation in the *ALMS1* gene. Dilated cardiomyopathy (DCM) is one of the well-recognized features of the syndrome ranging from sudden-onset infantile DCM to adult-onset cardiomyopathy, sometimes of the restrictive hypertrophic form with a poor prognosis. We aimed to evaluate severe cardiomyopathy in Alström syndrome in infancy and display susceptible specific mutations of the disease, which may be linked to severe DCM. Secondarily we reviewed published mutations in *ALMS1* with cardiomyopathies in the literature.

**Method:**

We represent new mutagenic alleles related to severe cardiomyopathy and cardiac outcome in this patient cohort. We evaluated echocardiographic studies of nine Turkish patients diagnosed with Alström syndrome (between 2014 and 2020, at age two weeks to twenty years). Thus, we examined the cardiac manifestations of a single-centre prospective series of nine children with specific ALMS mutations and multisystem involvement. All patients underwent genetic and biochemical testing, electrocardiograms, and echocardiographic imaging to evaluate systolic strain with speckle tracking.

**Results:**

Four of the patients died from cardiomyopathy. Three patients (including three of the four fatalities) with the same mutation (c.7911dupC [p.Asn2638Glnfs*24]) had cardiomyopathy with intra-familial variability in the severity of cardiomyopathy. Global longitudinal strain, a measure of systolic contractile function, was abnormal in all patients that can be measured.

**Conclusion:**

Cardiac function in ALMS patients with infantile cardiomyopathy appears to have different clinical spectrums depending on the mutagenic allele. The c.7911dupC (p. Asn2638Glnfs*24) mutation can be related to severe cardiomyopathy. Parents can be informed and consulted about the progression of severe cardiomyopathy in a child carrying this mutagenic allele.

**Supplementary Information:**

The online version contains supplementary material available at 10.1186/s13023-022-02483-7.

## Introduction

Alström syndrome (ALMS) (OMIM# 203800) is a rare autosomal recessive genetic disorder [1]. About 800 individuals diagnosed with Alström syndrome have been identified worldwide. It is caused by mutations in the ALMS1 gene located on chromosome 2p13, which encodes a predicted 461.2-kDa protein of 4169 amino acids. The functions of this protein include microtubule organization, particularly the formation and maintenance of cilia [2]. The metabolic abnormalities include severe insulin resistance, type 2 diabetes (T2D), hypertriglyceridemia, thyroid, and hepatic dysfunction.

Dilated cardiomyopathy occurs in over 60% of patients with ALMS at different life periods. The onset and progression of the cardiomyopathy show intra-familial variability. More than 40% of infants with ALMS present severe dilated cardiomyopathy, although this is reported as improving after infancy [3, 4]. In about 20% of Alström syndrome cases, restrictive cardiomyopathy with fibrosis and pulmonary hypertension develops during adolescence or adulthood. Diffuse interstitial myocardial fibrosis in ALMS is associated with left ventricular (LV) systolic function [5–8].

Hence, ALMS is a complex multisystem disorder; particular mutations may explain the variability of clinical presentations. The mutations related to the severity of cardiomyopathy have not been defined yet. Here, we represent a family with multiple affected members with ALMS with a highly deleterious mutation associated with severe infantile cardiomyopathy and cardiac outcome in Alström syndrome patients. We aimed to evaluate severe cardiomyopathy in Alström syndrome in infancy and to display susceptible specific mutations of the disease, which may be linked to severe DCM. Secondarily we reviewed published mutations of ALMS with cardiomyopathies in the literature.

## Materials and methods

**Fig. 1:**
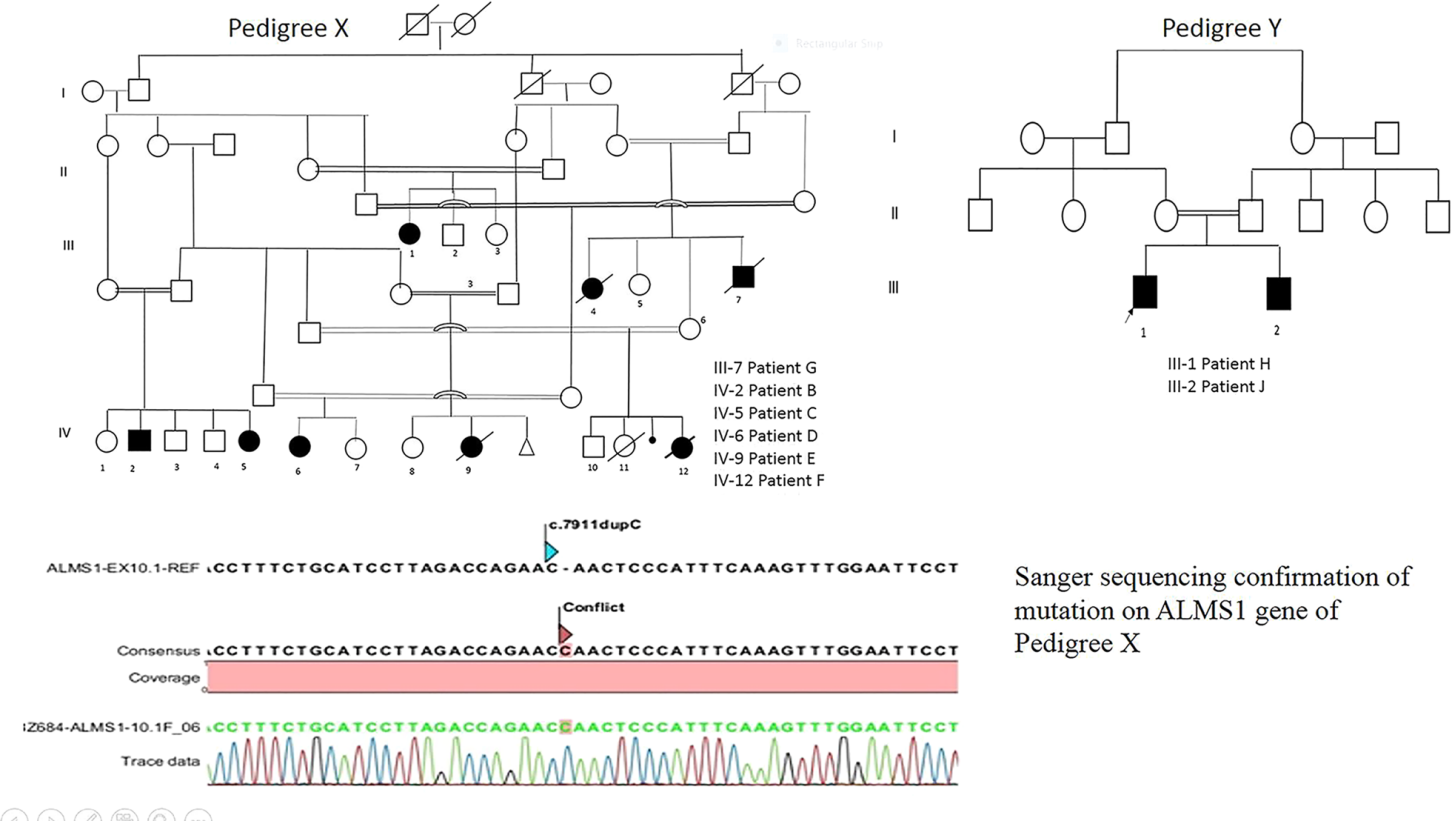
Pedigree of the patients, Sanger sequencing confirmation of mutation on *ALMS1* gene

Between March 2014 and November 2020, we evaluated the echocardiographic studies of nine Turkish patients diagnosed with ALMS (Table 1, Additional file 1: Table S1). All the patients had mutations in the ALMS1 gene (Fig. 1. Pedigree X and Y).

**Table 1:** Echocardiographic measurements in nine children with Alström syndrome

	No of patients	Mean ± SD (min–max)	Reference values*
*LV systolic functions*			
EF with Teicholz formula (%)	9	42.5 ± 43 (24–58)	> 55
Speckle strain (GLS) (%)	5	− 12.74 ± 4.06 (− 8–16)	− 17 − 24
*RV systolic functions*			
FAC (%)	9	37 ± 7 (26–47)	> 35
TAPSE (mm)	9	13 ± 4.6 (6–18)	> 18

*EF* Ejection fraction; *LV* left ventricular; *RV* right ventricular, *RV FAC* right ventricular fractional area change; *TAPSE* tricuspid annular plane systolic excursion, *GLS* global longitudinal strain

^*^Recommendations for quantification methods during the performance of a pediatric echocardiogram: a report from the Pediatric Measurements Writing Group of the American Society of Echocardiography Pediatric and Congenital Heart Disease Council. J Am Soc Echocardiogr 2010; 23: 465–95

We measured kidney and liver function tests, haemoglobin A1c (HbA1c), and lipid panels for all the patients and performed standard 12-lead electrocardiograms (ECGs). Cardiac measurements were performed according to the American Society of Echocardiography (ASE) guidelines [9].

We calculated LV ejection and shortness fractions (LVEF and LVSF, respectively) using the Teicholz formula [9]. We analyzed right ventricular (RV) functions with RV fractional area change (FAC) and Tricuspid annular plane systolic excursion (TAPSE).

Further analysis of global myocardial function was performed with Speckle tracking echocardiography (GLS). Cardiac magnetic resonance imaging (CMR) imaging studies were performed on two patients (Patients A and C) to assess cardiac anatomy.

The ALMS1 gene was sequenced for each patient after the clinical diagnosis, including all exons and exon–intron boundaries, using next-generation sequencing on the Illumina Miseq platform. Lipid panel, blood glucose, insulin, and thyroid function tests were collected. ECG verified eye findings. Tympanometry and distortion product otoacoustic emissions (DPOE) testing evaluated the hearing situation (Table 2).

**Table 2:** Clinical features in patients with Alström syndrome

Patient No	Weight (kg)	Obesity	Vision Loss	Hearing loss	Homa-IR	Hyperlipidemia (mg/dL)	Treatment	Other	EF % 1	EF % 2	
A	62	+	+	+	12.7	+	Enalapril Furosemid	Acanthosis Nigrikans, nephrocalcinosis	40	41	Ex 19 y
B	64	+	+	+			Enalapril Furosemid Spironolakton	Scoliosis	26	48	
C	17	+	+	−			Kaptopril Furosemid Digoxin		42	43	43
D	18	+	+	−	1.58	−	Captopril		59	55	
E	6	−	+	−			Enalapril Furosemid Spironolakton Digoksin Aspirin Metacartin		43	39	Ex in infancy
F	7	+	+	−			Digoxin Captopril Furosemid Spironolakton		25	35	Ex infancy
G	79	+	+	+			Enapril		59	58	Ex 17 y
H	72	+	+	+			Enapril		45	55	
J	11	−	+	−			Levosimendan Kaptopril, Spironolakton		40	29	

## Results

The demographic and clinical characteristics of the patients are displayed in Table 2. Eight of the patients (8/9) had been diagnosed with cardiomyopathy. Six patients had infancy onset cardiomyopathy, while the other two had been diagnosed after infancy/ adolescence or early adulthood. Two patients of the six infantile-onset cardiomyopathies with the same mutation died before two years old, at 16 months and 22 months, respectively. Three patients with adolescence-onset CMP responded partially to heart failure medications. However, their recent ejection fractions were still at the lower end of the normal range in two and deteriorated in one patient. In 2 families with multiple affected children (Families X, Y), the siblings had similar spectrums for cardiomyopathy; in Family X, the older patient had mild cardiomyopathy while his sister, who was monitored by echocardiograms biannually since birth, displayed severe infantile cardiomyopathy until the age of two then improved with heart failure treatment to a mild form of cardiomyopathy.

Five patients were clinically stable at the evaluation time, without symptoms or signs of heart failure. There was no significant resting tachycardia and normal blood pressure. All patients received beta-blockers and angiotensin-converting enzyme inhibitors. The ECG was normal in 3 patients and abnormal in 2 patients. Abnormalities included left ventricular hypertrophy (n = 1), left axis deviation (n = 3), non-specific T wave changes (n = 3), right axis deviation (n = 1), second-degree atrioventricular block (n = 1). and the ECG was otherwise normal.

Among all patients, we found left ventricular dilatation or systolic dysfunction in all patients (Table 1). Valvar involvement was also noted in two patients with thickened mitral valve leaflets and moderate mitral regurgitation. One patient had restrictive cardiomyopathy, and another showed prominent LV trabeculations with deep intertrabecular recesses filled with blood from the ventricular cavity. Hypertrabeculation with multiple crypts at the apical postero-lateral aspects of the left ventricle. Patients' detailed individual echocardiographic values were presented in Additional file 1: Table S1.

**Fig. 2:**
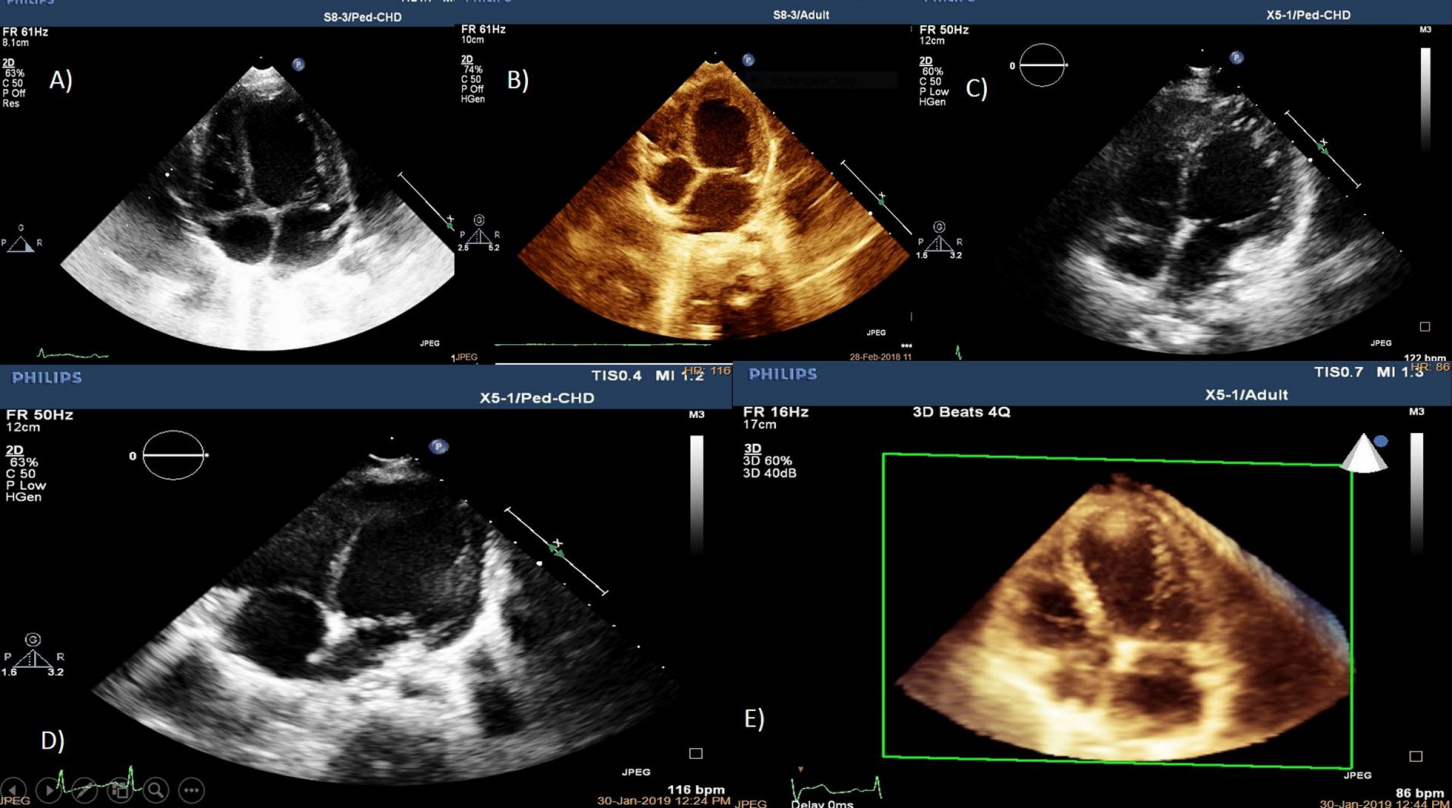
Left ventricular dilatation of patients with Alström syndrome on echocardiography. **a** Patient C, **b** Patient B, **c** Patient C, **d** Patient E, **e** Patient F

CMR was performed on two children and had evidence of decreased left ventricular function. Strain parameters of the left ventricle, including GLS, were calculated in three available patients. GLS of the left ventricle was abnormal in three children (Fig. 2).

### Genetic testing

ALMS1 whole gene sequencing was performed for all nine patients in this study (Table 3, Fig. 2). We detected a homozygous single nucleotide duplication on exon 10 of the ALMS1 gene (LRG_741t1[A1] c.7911dupC (p. Asn2638Glnfs*24) as previously reported by Marshall et al. [8]. This mutation is predicted to form a truncated protein, thus being highly deleterious and possibly responsible for the severe phenotype. A parental segregation study by Sanger sequencing confirmed the mutation (Table 4).

**Table 3:** Confirmed mutations of Patients

Pt No	Age at diagnosis	Consanguinity	Zygosity	*ALMS1* mutations in clinical Alström
A	14 y	+	Homozygote	*ALMS 1* gene
B_#¶_	16 y	+	Homozygote	c.7911dup C (p. Asn2638Glnfs*24)
C^#¶^	1 y	+	Homozygote	c.7911dup C (p. Asn2638Glnfs*24)
D^¶^	3 y	+	Homozygote	c.7911dup C (p. Asn2638Glnfs*24)
E^¶^	10 m	+	Homozygote	c.7911dup C (p. Asn2638Glnfs*24)
F^¶^	1 m	+	Homozygote	c.7911dup C (p. Asn2638Glnfs*24)
G	10 y	+	Homozygote	c.7905-7906InsC (p. N2636Qfs*24)
H^#^	3 m	+	Homozygote	c.7316C > A (p.Ser2439*)
J^#^	1.5 m	+	Homozygote	c.7316C > A (p.Ser2439*)

^#^ Siblings

^¶^Cousins

**Table 4:** Review of published mutations related with cardiomyopathy in Alström patients

Article				CMP onset			
	Mutation	Patient no	Type	İnfant	Adult	Prognosis	Outcome
JL Michaud / 1996 The Journal of Pediatrics (12)	NA	5	DCM	5		EF normal (1–10 y)	
JD Marshall et al./2005 Arch Intern Med (4)	Mutations in at least 1 allele of ALMS1 69 2p13-specific haplotypes,n 36	112	DCM	79 (1–16 m)	24 (5–36 y)		10 pt died
JC Smith et al. /2007 Eur J Hum Genet (23)	Pathogenic mutation in the ALMS1 gene in 8 pt	15	DCM	4	3		NA
Ozantürk A /2015 Journal of Human Genetics (25)	c.4156insAp.Thr1386Asnfs*15 c.5311C > Tp.Gln1769* c.5969C > Gp.Ser1990* c.9749C > A c.12117 + 20delT (IVS19-8delT)p.Ser3250* c.11055ins(n)331 c.11870-3 T > Gp.Val3958fs*			8	8		
A Brofferio et al./2017 Molecular Genetics and Metabolism (11)	1 c.11316_11319delAGAG p.Glu3773Trpfs*18 c.11416CNT p.Arg3806* 3 c.10535GNA p.Trp3512* c.11291GNA p.Ser 3764* 5 c.4156dupA p.Thr1386Asnfs*15 6 c.10775delC p.Thr3592Lysfs*6 c.2234CNG p.Ser 745* 8 c.10775delC p.Thr3592Lysfs*6 c.10775delC p.Thr3592Lysfs*6 10 c.10775delC p.Thr3592Lysfs*6 c.10775delC p.Thr3592Lysfs*6 15 c.4885CNT p.Gln1629* c. 5923 CNT p.Gln 1975* 16 c.592CNT p.Gln198* c.1610_1611delTC p.Leu538Glnfs22 20 c.10849GNT p.Glu3617* c.10483CNT p.Gln3495* 23 c.11314dupA p.Arg3772Trpfs*10 c.10885CNT p.Arg3629* c.1903CNT p.Gln635* c.3579CNG p.Tyr1193*	38		10			
	13 c.10539_10557ins(n)19 p.Lys3545Asnfs*18 c.10539_10557ins(n)19 p.Lys3545Asnfs*18 19 c.6305CNA p.Ser2102* c.10775delC p.Thr3592Lysfs*6 21 c.10775delC p.Thr3592Lysfs*6 c.3716_3719del p.Ser1240Thrfs*23 24 c.5311CNT p.Gln1769* c.5311CNT p.Gln1769* 25 c.11651_11652insGTTA p.Asn3885LeufsX9 c.4817delA p.Lys1608ArgfsX9				5		NA
SA Hollander/2017 Am J Med Genet. (21)	First variant (c.2816 T > A; p.Leu939*) Second variant (c.10837_10838delCA; p.Gln3613Alafs*2)	2	DCM	+			
Nerakh G/ 2019 The Indian Journal of Pediatrics (139	Homozygous nonsense variant c.2816 T > A (p.Leu939Ter)	2	DCM	+		NA	
Mahamid j /2013 Pediatr Cardiol **(14)**	Homozygous for a nonsense mutation in exon 10 of *ALMS1*, c.8008C > T (p.R2637X)	2	DCM	+			
Curent study	c.7911dup C (p. Asn2638Glnfs*24) (6 patient) Homozygote c.7905–7906 Ins C (p. N2636Qfs*24) (1patient) Homozygote c.7316C > A (p.Ser2439*) (2 patient)	9	DCM 8 Restrictive CMP 1	5			5 died (4 with the same mutation)

### The outcome of patients with c.7911dupC (p. Asn2638Glnfs*24) mutation

Patients in Pedigree X had similar onset, progression, and outcome of the disease processes of Alström syndrome (Fig. 1). The dilated cardiomyopathy was reported in infancy, although it was evident soon after birth and progressed to death in two cases, with severe dilated and worsening cardiomyopathy, despite the anti-congestive medication. Several months later, the other four patients with the same mutation manifested dilated cardiomyopathy but survived beyond infancy. The seven-year-old and five-year-old girls had systolic dysfunction on echocardiography. One of them was determined to have had cardiomyopathy during infancy since he had systolic dysfunction (Fig. 2).

## Discussion

Here, we have represented severe infantile-onset cardiomyopathy in a family with a recently recognized mutation of ALMS1 [8]. Although genotype–phenotype correlation has not been demonstrated for a specific clinical spectrum for the individual mutations in ALMS1 in all reported articles on cardiomyopathies, we think that the c.7911dupC (p. Asn2638Glnfs*24) mutation may be one of the significant determinants of the severe cardiomyopathy.

The cohort of patients with ALMS has demonstrated two major clinical spectrums related to cardiomyopathy: infantile-onset (43%) and those with later onset (18%). Cardiomyopathy has not been diagnosed in the remaining patients (39%, ages 2–33 years). Patients with infantile-onset cardiomyopathy have had apparent recovery of cardiac function within three years [4]. Previous reports have suggested the complete recovery of infantile-onset cardiomyopathy in Alström syndrome patients [4].

Bond et al. observed seven families in which heart failure due to dilated cardiomyopathy had been presented as a symptom within the first three months. In that study, all patients responded to conventional supportive therapy, and the cardiomyopathy resolved spontaneously within six months. The authors suggested that dilated cardiomyopathy in ALMS presenting in the first year of life can regress completely [10].

Brofferio et al. suggested that cardiac involvement in ALMS is more common than previously reported. A third (13 of 38) of patients in their study had infantile cardiomyopathy, with EF results ranging from 40 to 66%. The authors proposed longitudinal follow-up studies to determine whether this mild cardiomyopathy progresses into a more significant disease in all Alström syndrome patients [11].

Due to the reported outcomes of cardiomyopathy in ALMS, most series report cardiac function with a history of infantile cardiomyopathy that improves within the first 2–3 years of life [12, 13]. However, we suggest that recovery of cardiac function is not characterized by time; instead, it is a matter of the type and characterization of the mutation. The severity of cardiomyopathy depends on the truncated protein, accordingly to the mutation. In our patient series with the ALMS, c.7911dupC (p. Asn2638Glnfs*24) mutation, cardiomyopathy was partially resolved in two cases. In contrast, two patients with the same mutation died despite maximal drug support. Similar to our results, Mahamid et al. [14] reported two brothers, 2 and 3 years of age, diagnosed with Alström syndrome during febrile respiratory infection, initially presenting in infancy with severe dilated cardiomyopathy. The disease course in the older sibling has mainly resolved while cardiomyopathy in the younger sibling deteriorated despite maximal support with heart failure medications.

Our findings of LV systolic and diastolic dysfunctions in all the children correspond with previous reports [15]. Phenotypic variation, such as differences in severity of cardiomyopathy among siblings with the same mutation, suggests that besides any variability due to the mutation itself, there is an interplay between multitudes of potential genetic modifiers, with environmental factors leading to the range in severity of the Alström syndrome phenotypic spectrum [16].

In genotype–phenotype correlation studies, researchers have found no association between the location or type of ALMS1 mutations and T2D, body mass index (BMI), or dilated cardiomyopathy [10, 17]. However, in another study, Ichihara et al. did identify a link between the polyglutamine repeat in ALMS and early onset myocardial infarct [18].

Despite advances in our knowledge of the spectrum of ALMS1 mutations, we still do not have enough evidence for prognostic predictions based on genotype. Histopathology of affected patients has shown diffuse interstitial fibrosis affecting the myocardium, later confirmed by cardiac MRI [5, 6]. Dilated cardiomyopathy (DCM) can occur suddenly in infancy (in the first months of life) due to aberrant differentiation of cardiomyocytes [19, 20]. Histologic findings of infantile cardiomyopathy have not been presented yet, but there are studies in which mitogenic cardiomyopathy is described in patients with Alström syndrome [21, 22].

Mitogenic cardiomyopathy is a very rare human phenotype. Shenje et al. showed homozygous or compound heterozygous mutations among six infants with Alström syndrome, suggesting mitogenic cardiomyopathy is the leading cause of lethal cardiomyopathy. They showed that ALMS1 is a key for cell cycle regulation in perinatal cardiomyocytes. They removed the hearts of each individual they studied, either at the time of transplantation due to end-stage heart failure or after death from heart failure [21]. Although we did not perform autopsies, mitogenic cardiomyopathy may be the cause of death of our patient with severe infantile cardiomyopathy. Restrictive cardiomyopathy develops slowly in adolescents and adults [23]. In line with previous reports, our first adolescent case (Patient A) died because of restrictive cardiomyopathy.

Although intra-familial differences observed in the disease presentation of our patients carrying the same mutation (Patients B, C, D, E, and F) complicated our notion about this recently recognized mutation, we suggest that there are mutations that tend to severe cardiomyopathy. The phenotypic differences observed among individuals within the same family may result from gene–gene, protein–protein interactions or functional polymorphisms [24]. Some authors have suggested that environmental and unknown genetic modifiers probably interact with ALMS1 [13] children growing up in the same family with similar environmental factors to support expression patterns of genotype. Further to the evidence from our patients, Hollander et al. showed that the clinical course of Alström syndrome might vary concerning cardiac disease manifestations, even in monozygotic twins [25].

In a literature review of 44 patients from our country by Ozanturk et al., the authors reported death of two ALMS patients with an unknown genetic mutation might be linked to CMP [26]. Our patients had not been displayed in that study. In our ALMS cohort, cardiomyopathy appeared to be a primary manifestation.

In a study by Brofferio et al., there were subclinical strain echocardiographic abnormalities in nearly all patients in their cohort, suggesting the presence of myocardial disease in patients without overt cardiomyopathy, including those with recovered from infantile cardiomyopathy [11]. However, the individuals in the current cohort also displayed significant metabolic disturbances, including T2D, vision and hearing loss, elevated triglyceride and cholesterol levels, and obesity. These metabolic disturbances can themselves be substantial cardiac risk factors over time. Our findings of abnormal GLS with LV systolic dysfunction and restrictive cardiomyopathy of both ventricles support their results.

In another study, while mild interstitial myocardial fibrosis was present in patients without a prior history of dilated cardiomyopathy, moderate-to-severe interstitial fibrosis was found in ALMS patients with dilated cardiomyopathy [4]. Progressive fibrosis and cardiac risk factors (obesity, T2DM) can explain the restrictive cardiomyopathy of our case (Patient A), diagnosed in adolescence with restrictive cardiomyopathy.

## Conclusion

Early diagnosis of ALMS and genetic testing provide important prognostic information to the paediatric cardiologist and enable the family to consider likely outcomes. First, appreciation of the aetiology of cardiomyopathy can guide appropriate management plans. Second, determining the exact causal mutation(s) in the index patient can clarify the inheritance pattern. Thus, families can be consulted on the recurrence risk in future offspring and seek prenatal testing in subsequent pregnancies.

Our comprehensive evaluation of nine children with Alström syndrome suggests that cardiac function in ALMS patients with infantile cardiomyopathy has different clinical spectrums depending on the mutagenic allele. We recommend that the c.7911dupC (p. Asn2638Glnfs*24) mutation can be related to severe cardiomyopathy.

## Supplementary Information

**Additional file 1. Table S1. **Detailed individual echocardiographic studies of nine Turkish patients diagnosed with ALMS.

## Data Availability

Please contact author for data requests.
