# Supplementary material for: Mutation identification and prediction for severe cardiomyopathy in Alström syndrome, and review of the literature for cardiomyopathy
Source: Orphanet J Rare Dis. 2022 Sep 15;17:359. doi: 10.1186/s13023-022-02483-7 (PMC9479229; doi:10.1186/s13023-022-02483-7)
Supplement: Supplementary file 1 — Additional file 1. Table S1. Detailed individual echocardiographic studies of nine Turkish patients diagnosed with ALMS. [file 13023_2022_2483_MOESM1_ESM.docx]

|  | Age at last examination | LV systolic functions | | | LV diastolic functions | | | | RV systolic functions | | Type of CMP |
| --- | --- | --- | --- | --- | --- | --- | --- | --- | --- | --- | --- |
|  |  | Teicholz formula (%) last examination | Speckle strain (%) | | Pulsed Doppler  (cm/s) | | | Tissue Doppler | FAC  (%) | TAPSE (mm) |  |
|  |  | EF | Total GLS | | e | a | e/a | e/e' |  |  |  |
| A | 17 y | 24 |  | | 67 | 42 | 1.6 | 5.8 (5.4–9.3) | 26 | 12 | Restrictive CMP |
| B | 17 y | 48 | -18 | -10^#^ | 84 | 35 | 2.38 | 6.28 (5.4–9.3) | 40 | 15 | DCM |
| C | 5 y | 43 | -13.2 | | 74 | 67 | 1.1 | 6.3 (5.3–9.4) | 38 | 17 | DCM |
| D | 7 y | 55 | -15 | | 121 | 63 | 1.93 | 8.1 (5.3–9.4) | 45 | 17 | DCM |
| E | m | 36 | -8 | | 54 | 65 | 0.83 | 14 (6.8–14) | 30 | 7 | DCM |
| F | 22 m | 35 | -9.5 | | 61 | 51 | 1.1 | 15.9 (6.8–14) | 30 | 6 | DCM |
| G | 20 y | 58 |  | | 74 | 36 | 1.5 | 5.2 (5.4–9.3) | 47 | 18 | DCM |
| H | 16 y | 55 |  | | 85 | 66 | 1.3 | 7.1 (5.4–9.3) | 43 | 17 | DCM |
| J | 18 m | 29 |  | | 64 | 61 | 1.1 | 12 (6.8–14) | 34 | 10 | DCM |

Supplementary Table 1. Echocardiographic parameters in nine children with Alström syndrome.

RV: right ventricular, RV FAC: right ventricular fractional area change; TAPSE: tricuspid annular plane systolic excursion (normal value > 18 mm). The early, rapid filling phase of diastole is represented by the e-wave. Atrial contraction occurs in late diastole and is represented by the a-wave. The early diastolic tissue Doppler velocity, commonly denoted as e’, represents RV relaxation. Increased e/e’ ratios represent increased RV filling pressures. Normal values for children at 5.5–9 have recently been published. GLS: global longitudinal strain (normal values between −17 % and −24 %). ^#^Value at last visit.
